# The E3 Ubiquitin Ligase CRL5 Regulates Dentate Gyrus Morphogenesis, Adult Neurogenesis, and Animal Behavior

**DOI:** 10.3389/fnins.2022.908719

**Published:** 2022-06-21

**Authors:** Raenier V. Reyes, Keiko Hino, Cesar Patricio Canales, Eamonn James Dickson, Anna La Torre, Sergi Simó

**Affiliations:** ^1^Department of Cell Biology and Human Anatomy, University of California, Davis, Davis, CA, United States; ^2^Department of Physiology and Membrane Biology, University of California, Davis, Davis, CA, United States

**Keywords:** CRL5, RBX2, adult neurogenesis, mossy fibers, dentate gyrus development

## Abstract

The dentate gyrus (DG) is an essential part of the hippocampal formation and participates in the majority of hippocampal functions. The DG is also one of the few structures in the mammalian central nervous system that produces adult-born neurons and, in humans, alterations in adult neurogenesis are associated with stress and depression. Given the importance of DG in hippocampal function, it is imperative to understand the molecular mechanisms driving DG development and homeostasis. The E3 ubiquitin ligase Cullin-5/RBX2 (CRL5) is a multiprotein complex involved in neuron migration and localization in the nervous system, but its role during development and in the adult DG remain elusive. Here, we show that CRL5 participates in mossy fiber pruning, DG layering, adult neurogenesis, and overall physical activity in mice. During DG development, RBX2 depletion causes an overextension of the DG mossy fiber infrapyramidal bundle (IPB). We further demonstrate that the increased activity in Reelin/DAB1 or ARF6 signaling, observed in RBX2 knockout mice, is not responsible for the lack of IPB pruning. Knocking out RBX2 also affects granule cell and neural progenitor localization and these defects were rescued by downregulating the Reelin/DAB1 signaling. Finally, we show that absence of RBX2 increases the number neural progenitors and adult neurogenesis. Importantly, RBX2 knockout mice exhibit higher levels of physical activity, uncovering a potential mechanism responsible for the increased adult neurogenesis in the RBX2 mutant DG. Overall, we present evidence of CRL5 regulating mossy fiber pruning and layering during development and opposing adult neurogenesis in the adult DG.

## Introduction

Dentate gyrus (DG) morphogenesis is a complex process that requires the coordination of neural stem cell (NSC) and intermediate progenitor (IP) proliferation, neurogenesis, and cell migration to form the well-known arrowhead, laminated structure within the hippocampus (Khalaf-Nazzal and Francis, 2013; Nelson et al., 2020). The principal neurons in the DG are granules cells (GCs), located in the granule cell layer (GCL), and IPs and NSCs below the GCs in the subgranular zone (SGZ). Importantly, the NSCs produce adult-born GCs throughout the lifespan of many animals (Goncalves et al., 2016). In humans, defects in DG development or homeostasis cause a variety of diseases, including epilepsy and mood affective disorders (Hayashi et al., 2018; Santos et al., 2019).

The murine DG development starts at embryonic day (E) 13 by the first generation of immature GCs, IPs, and NSCs in the ventricular zone area known as dentate notch (i.e., Primary germinative matrix). This mix of post-mitotic and progenitor cells migrate, and divide, in their way to the DG primordium and migrate around the hippocampal fissure to stablish the upper blade of the DG, first, and the lower blade, afterward. Finally, a tertiary germinative matrix located in the hilar area will generate GC for the inner leaflet of the GCL and the NSCs for the SGZ (Altman and Bayer, 1990; Khalaf-Nazzal and Francis, 2013; Hayashi et al., 2015; Nelson et al., 2020).

In comparison to the cortex, fewer signaling pathways have been studied in the context of DG development. Among the few, the Reelin/DAB1 signaling pathway has been shown to be indispensable for migration directionality of GC, and likely of IPs and NSCs, at late DG migratory stages (Li et al., 2009; Wang et al., 2018). Importantly, a combination of Reelin/DAB1 and Notch signaling is necessary for radial glia scaffolding, contributing to proper cell migration and DG development (Sibbe et al., 2009; Brunne et al., 2013). Moreover, Reelin/DAB1 signaling positively correlates with adult neurogenesis in gain- and loss-of-function mouse models (Pujadas et al., 2010; Teixeira et al., 2012).

The E3 ubiquitin ligase Cullin-5/RING ligase (CRL5) is a multiprotein complex nucleated around the core proteins Cullin-5 (Cul5) and RING box protein 2 (RBX2; also known as RNF7). CRL5 uses up to 38 different substrate adaptors to recruit target proteins to the complex for ubiquitylation (Okumura et al., 2016). In the central nervous system, CRL5 contributes to neuron migration and localization, neuronal layering, and dendritogenesis (Simo and Cooper, 2013; Fairchild et al., 2018; Han et al., 2020). Two CRL5-regulated signaling pathways are mainly associated with these phenotypes, the Reelin/DAB1 and ARL4C/ARF6 signaling. Knocking out of RBX2 causes ectopic cortical layering and the accumulation of signaling effectors and among them the active, tyrosine-phosphorylated DAB1 (pY-DAB1). Reduction of DAB1 accumulation partially rescues the cortical layering phenotypes caused by RBX2 depletion. Moreover, knocking out SOCS7, a CRL5 substrate adaptor that binds and recruits pY-DAB1 for poly-ubiquitylation, causes a similar pY-DAB1 accumulation and cortical disruption as RBX2 depletion without affecting other CRL5-dependent signaling effectors (Simo and Cooper, 2013; Han et al., 2020). In the hippocampus, CRL5 also regulates neuron polarity and dendritogenesis by opposing the activity of the small GTPases ARL4C and ARF6 (Hofmann et al., 2007; Han et al., 2020). However, the role of CRL5 in DG development and adult neurogenesis remains elusive. Here, we show that CRL5 participates in developmental GC axon (i.e., mossy fibers) pruning, independently of Reelin/DAB1 or ARF6 activity. Moreover, CRL5 controls the number of NSCs and the lamination of GCs, IPs, and NSCs, in part through the downregulation of Reelin/DAB1 signaling. Finally, we show that CRL5 regulates adult neurogenesis, likely by promoting higher levels of physical activity.

## Methods

### Animals

All animals were used with the approval from the University of California, Davis Institutional Animal Care and Use Committees and housed in accordance with the guidelines provided by the National Institute of Health. Control (*rbx2 fl/fl*), Rbx2cKO-Emx1 (*rbx2 fl/fl; Emx1-Cre*), *dab1* and *socs7* knockout mice were obtained as described in Simo and Cooper (2013); Fairchild et al. (2018), and Han et al. (2020). To generate the RBX2; NestinCREERT2; Ai9 mice, me crossed *rbx2 fl/fl* mice with the Cre-reporter Ai9 mice [*Gt(ROSA)26Sor^TM 9(CAG–tdTomato)^*^Hze^, The Jackson Laboratory #7909; Madisen et al., 2010] until homozygosis for both genes. We also crossed RBX2 floxed mice with *Nestin-Cre/ERT2* transgenic mice (The Jackson Laboratory #16261; Lagace et al., 2007) to obtain *rbx2 fl/*+; *Nestin-Cre/ERT2* mice. Finally, we intercrossed *rbx2 fl/fl; Ai9/Ai9* mice with *rbx2 fl/*+; *Nestin-Cre/ERT2* mice to obtain control (*rbx2 fl/*+; *Ai9/*+; *Nestin-Cre/ERT2*) and tamoxifen-induced RBX2 knockout NSCs (*rbx2 fl/fl; Ai9/*+; *Nestin-Cre/ERT2*). To generate double RBX2 and ARF6 conditional knockout mice, we obtained Arf6 floxed mice (*Arf6^tm1.1Gdp^*, The Jackson Laboratory #28669; Marquer et al., 2016), and crossed it with Rbx2cKO-Emx1 mice until homozygosis of *rbx2* and *arf6* floxed alleles and heterozygosis of the Emx1-Cre allele. When embryonic samples were required, females were mated, and the morning a vaginal plug was observed was considered P0.

### Histology and Immunofluorescence

Postnatal (P) 21 and P75 mice were anesthetized and transcardially perfused with phosphate-buffered saline (PBS) followed by 3.7% formalin/PBS using a peristaltic pump. Perfused brains were collected and postfixed at 4°C overnight in the same solution. Tissues were cryoprotected with 30% sucrose/PBS solution. Next, brains were embedded in Optimum Cutting Temperature (OCT) compound (Tissue-Tek) and quickly frozen using dry-ice. OCT-embedded brain blocks were cryo-sectioned on a coronal plane (30 μm). Immunostainings were performed in free-floating sections with agitation. First, sections were antigen retrieved with 10 mM sodium citrate (pH 6) at 95°C for 20’. Then, tissue was blocked with PBS, 0.5% Triton X-100, and 5% milk or 10% normal donkey serum for 1 h at room temperature. Blocking solution, but reducing Triton X-100 concentration to 0.3%, was used for primary antibody incubation (overnight, 4°C). The following primary antibodies were used for immunohistochemistry: anti-Calbindin (1/10; NeuroMab #73-452), anti-Calbindin (1/200; Sigma-Aldrich #C9848), TUJ1 (1/500; Biolengend #801201), anti-DCX (1/200; Santa Cruz Biotechnology #sc-8066, discontinued), anti-SOX2 (1/200; Santa Cruz Biotechnology #sc-17320, discontinued); anti-Ki-67 (1/200; Biolegend #151202); anti-DAB1 (1/200; Sigma-Aldrich #HPA052033). Species-specific Alexa Fluor 488- and/or 568-conjugated immunoglobulin G (IgG) (1/200; Life Technologies) were used in blocking solution but reducing Triton X-100 concentration to 0.3% (90 min, room temperature). DAPI (Sigma-Aldrich) was used for nuclear staining. Images were taken in a Fluoview FV3000 confocal microscope (Olympus) or Axio Imager.M2 with Apotome.2 microscope system (Zeiss). All images were assembled by using FIJI and Photoshop and Illustrator (Adobe).

### Infrapyramidal Bundle Length Measurement

We measured the length of the IPB and *stratum pyramidale* of 3 brain slices, 100 μm apart, per brain, normalized the IPB length to the length of the *stratum pyramidale* in each slice, and average the results to obtain the normalized IPB length per brain. For consistency, we only measure IPB length in brain slices containing dorsal (septal) hippocampi.

### Dentate Gyrus Explants

DG explants were obtained from E18 control and RBX2cKO-Emx1 brains using a previously published protocol (Gil and del Rio, 2012). DG explants were co-culture with HEK293T cell aggregates expressing mock (pCAG-EGFP) or Semaphorin-3F expression plasmid in three dimension collagen (Thermo Fisher Scientific, #A1048301) matrices for 96 h. Afterward, explants were fixed and immunostained using an anti-β-III-Tubulin antibody (1/200, Biolegend #801201) and Alexa Fluor-488 secondary antibody (1:200; Life Technologies). Images were taken in a Fluoview FV3000 confocal microscope (Olympus). To quantify axonal growth each explant was divided into four quadrants and the number of axons that crossed a line placed at a distance of 100 μm from the limit of the explant was counted for the proximal and distal quadrants. The proximal/distal (P/D) ratio of axonal growth was obtained by dividing the β-III-tubulin fluorescent signal intensity in the proximal quadrant by that in the distal quadrant; yields 1 for radial growth, more than 1 for attractive effect and less than 1 for repulsive effect.

### RAC1/CDC42/RHO Pull Down Assay

The hippocampal regions of control or RBX2cKO–Emx1 mice (P21) were carefully dissected and lysed in lysis buffer (50 mM HEPES, 150 mM NaCl, 1.5 MgCl_2_, 1 mM EGTA, 10% Glycerol, 1% Triton X-100, and a protease and phosphatase inhibitors). Protein lysate were clear out of cellular debris by centrifugation. Protein supernatant were mixed with purified fusion proteins containing GST-hPAK1-PBD (RAC1 and CDC42 pull down) or GST-Rhotekin-RBD (RHO pull down) for 3 h at 4°C (de Rooij and Bos, 1997). GST-fused proteins and associated small GTPases were pull-down using Glutathione-Sepharose beads (Santa Cruz Biotechnology, #sc-2003). Beads were washed four times with cold lysis buffer and samples were resolved by SDS-polyacrylamide gels. Western blot analysis was performed as described in Han et al. (2020) using anti-RAC1, anti-CDC42, and anti-RHO antibodies (1/1,000, Santa Cruz Biotechnology, #sc-514583, #sc-390210, and #sc-418, respectively) to detect pulled down proteins and RAC1 in whole lysates. Pull down constructs were a generous gift from Dr. Jonathan Chernoff (Addgene plasmid #12217) and Dr. Martin Schwartz (Addgene plasmid #15247, Ren et al., 1999).

### Protein Analysis

P10 hippocampal samples from control (RBX2 fl/fl), Rbx2cKO-Emx1, SOCS7 +/–, and SOCS7 −⁣/⁣− mice were lysed in lysis buffer, resolved in SDS-polyacrylamide gels, and analyzed by Western blotting, as previously described. Blots were probed with anti-phosphotyrosine 4G10 (1/5,000; Millipore #05-321), then stripped and reprobe for DAB1 protein (1/5,000; Rockland #100-401-225).

### Open Fields Test

The open field test was performed and analyzed as described elsewhere (Silverman et al., 2011). Briefly, individual mice were placed in a VersaMax Animal Activity Monitoring System (AccuScan Instruments, Columbus, OH, United States) for a 30-min test session. The testing room was illuminated with overhead lighting at ∼ 30 lx. The chambers consisted of clear Plexiglas sides and floor, approximately 40 × 40 × 30.5 cm. Mice were placed in the center of the open field at the initiation of the testing session. Photocells at standard heights for recording activity were aligned 8 to a side, dividing the chamber into 64 equal squares. Each time an animal crossed a photoelectric beam it counted as an “event”. Horizontal activity (events), total distance (cm), vertical activity (events), and center time (sec.) were automatically collected using the Versamax activity monitor and analyzer software system. Test chambers were cleaned with 70% ethanol between test subjects. At least 5 min between cleaning and the start of the next session was allowed for ethanol evaporation and odor dissipation.

### Tamoxifen Injection and tdTomato + cells Quantification

P30 *rbx2 fl/*+; *Ai9/*+; *Nestin-Cre/ERT2* and *rbx2 fl/fl; Ai9/*+; *Nestin-Cre/ERT2* mice were intraperitoneally injected with 80 mg/kg body weight of Tamoxifen, dissolved in 1:20 solution of EtOH and corn oil (Feil et al., 2009), for 5 consecutive days. 30 days after the last injection, animals were transcardially perfused and processed for cryosection as described. Brains were section at 30 μm and all slices containing hippocampus were collected and mounted in stereological fashion. Brain slices were counterstained with DAPI and images of the whole DG in both hemispheres were taken with an Axio Imager.M2 with Apotome.2 microscope system (Zeiss). We quantified all *Ai9*+ cells in both DG per brain. *Ai9*+ cells outside the DG proper were not quantified.

### EdU Injection and Granule Cell Survival

P21 control and Rbx2cKO-Emx1 mice were injected with 12.5 mg/kg body weight of EdU (Click-iT EdU Imaging Kit, Life Technologies). 6 months post-injection, animals were processed as described and EdU detected following the manufacturer instructions.

### Statistics

Statistical analyses were performed with Prism 9 (GraphPad Software). Statistical analysis used for each experiment is described in the corresponding figure legend. For parametric sample distribution, unpaired Student’s *t*-test was used for two-population comparison and one-way ANOVA with Tukey’s *post-hoc* test for multiple comparisons. For the open field test result analysis, we used two-way ANOVA Tukey’s *post-hoc* test for multiple comparisons, except for the total activity were an unpaired Student’s *t*-test was used. For non-parametric sample distribution, Mann Whitney test was used for two-population comparison.

## Results

### CRL5 Promotes Mossy Fiber Pruning in the Developing Dentate Gyrus

**FIGURE 1:**
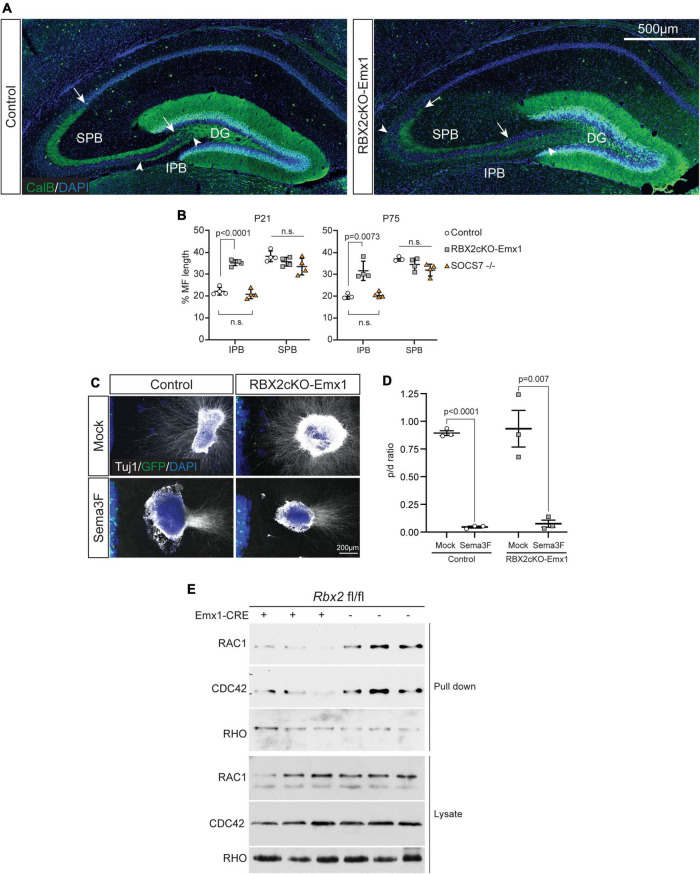
RBX2 participates in mossy fiber IPB pruning. **(A)** IPB overextension in the RBX2cKO-Emx1 mice at P21. Stainings of control and RBX2cKO-Emx1 DG showed a similar extension of mossy fiber SPB (arrows demarcate SPB extension), whereas mossy fiber IPD was ectopically extended in the RBX2 mutant DG (arrowheads demarcate IPB extension). **(B)** Quantification of IPB extension in control, RBX2cKO-Emx1, and SOCS7 knock out (–/–) DG at P21 (left) and P75 (right). Mean ± SEM. Statistics, one-way ANOVA with Tukey’s method adjusted *p*-value. **(C)** Axons from E18.5 control and RBX2 mutant DG explant were repelled when confronted with a source of Semphorin-3F (Sema3F). Notice the HEK293T cell aggregate transfected with control plasmid or Sema3F-expressing plasmid on the left hand side of the image. For quantification, DG explants were divided in four quadrants and β-III-Tubulin fluorescent signal measured in proximal (in front of the cell aggregate) and distal (opposite) quadrants. **(D)** The ratio of proximal and distal (p/d) showed that both control and Rbx2 mutant DG axons were repelled by Sema3F. Mean ± SEM. Statistics, unpaired Student’s *t*-test. **(E)** Decreased RAC1-GTP and CDC42-GTP levels upon RBX2 depletion in comparison to control. RHO-GTP levels remained unaffected.

To investigate the role of CRL5 during DG development, we conditionally depleted RBX2 in the telencephalon by intercrossing *rbx2* floxed mice with an *Emx1*-Cre driver mouse (Rbx2cKO-Emx1 mice), which readily depletes RBX2 and disrupts CRL5 activity from embryonic day (E)10 (Gorski et al., 2002; Han et al., 2020). Rbx2cKO-Emx1 mice are born at expected Mendelian rations, thrive as control littermates (*rbx2 fl/fl*), and survive until adulthood. To assess gross DG morphological defects, we stained control and Rbx2cKO-Emx1 samples against calbindin (CalB), which labels mature GCs, including GC axons (i.e., mossy fibers), at postnatal day (P)21 (Figure 1A). As expected, control brains showed two CalB+ axon bundles departing the DG, the long suprapyramidal bundle (SPB), which forms the *stratum lucidum* in the CA3, and the short infrapyramidal bundle (IPB) that after exiting the DG rapidly crosses the *stratum pyramidale* and joins the SPB (Bagri et al., 2003; Khalaf-Nazzal and Francis, 2013). In comparison, depletion of RBX2 caused an overextension of the mossy fiber IPB, reaching the apex of the CA3 curvature (Figures 1A,B). During development, SPB and IPB mossy fibers initially extend above and below the *stratum pyramidale* of the CA3, respectively, and during postnatal stages the IPB prunes almost completely, cross the *stratum pyramidale*, and joins the SPB (Bagri et al., 2003). First, we tested whether the IPB extension defects observed in RBX2 mutant animals were a temporal delay in axon pruning. Control and RBX2 mutant DG were collected at P75, a stage when IPB pruning is long completed, and stained against CalB. Similar IPB overextension was present in Rbx2cKO-Emx1 brains, highlighting a novel role for CRL5 in IPB pruning (Figure 1B and Supplementary Figure 1A). We hypothesized that signaling pathways deregulated in the RBX2 mutant brain may be responsible for this phenotype. Thus, we assessed whether sustained Reelin/DAB1 or ARF6 activation were responsible for IPB overextension. To test the role of sustained Reelin/DAB1 signaling in IPB pruning, we used Rbx2cKO-Emx1; DAB1 +/– mice, which significantly reduces the accumulation of pY-DAB1, and SOCS7 knockout mice, which promotes pY-DAB1 accumulation without affecting other CRL5-regulated signaling effectors (Supplementary Figure 2; Simo and Cooper, 2013; Han et al., 2020). Accumulation of pY-DAB1 in the SOCS7 mutant DG was not sufficient to cause IPB overextension and reducing DAB1 levels in Rbx2cKO-Emx1 failed to rescue IPB overextension (Supplementary Figures 1B,C). Next, we generated a new mutant mouse conditionally targeting RBX2 and ARF6 using the Emx1-CRE driver (RBX2/ARF6cKO-Emx1) and analyzed its IPB. Depleting ARF6 did not rescue IPB overextension cause by CRL5 inactivation (Supplementary Figure 1D). Interestingly, in all the conditions where we observed IPB overextension, there was also a complementary thinning of the SPB, due to the absence of IPB axons bundling with the SPB.

IPB overextension was previously described in mutant animals for the Semaphorin-3F co-receptors Neuropilin-2 and Plexin-3A (Chen et al., 2000; Cheng et al., 2001). Furthermore, Bagri et al. (2003) showed that, at postnatal stages, Sempahorin-3F secretion from neuropeptide Y-expressing interneurons drives IPB mossy fiber pruning. Next, we assessed whether RBX2 mutant GC axons were able to sense and respond to Sempahorin-3F using an axon repulsion assay. DG explants were obtained from control and Rbx2cKO-Emx1 embryos at E18 and co-cultured with HEK293T cells expressing either a mock plasmid (pCAG-EGFP) or Semaphorin-3F-expressing plasmid. In comparison to mock plasmid, expression of Semaphorin-3F trigger a strong repulsion of GC axons in both control and RBX2 mutant DG explants, indicating that RBX2 mutant GC axons respond to Semophorin-3F as controls (Figures 1C,D). Despite we have not tested the expression of Semaphorin-3F in our mutant animals, interneurons are not targeted by the Emx1-Cre strain used in our experiments (Gorski et al., 2002) and therefore we do not expect any defects on interneuron genesis, localization, or maturation.

At the molecular level, IPB pruning depends on activation of the RAC-GAP protein β2-Chimaerin and, consequently, inactivation of the Rac small GTPase family (Riccomagno et al., 2012). Surprisingly, pull-down assays using the p21-binding domain (PBD) of PAK1 showed that in RBX2 mutant samples RAC1-GTP and Cdc42-GTP levels are lower than control, while the activity of the RHOA GTPase remain unaffected (Figure 1E). These data suggest that CRL5-dependent deregulation of RAC1 and/or CDC42 activity may be responsible for the lack of IPB pruning observed in the RBX2 mutant DG.

### CRL5 Regulates Dentate Gyrus Lamination and Adult Neurogenesis

**FIGURE 2:**
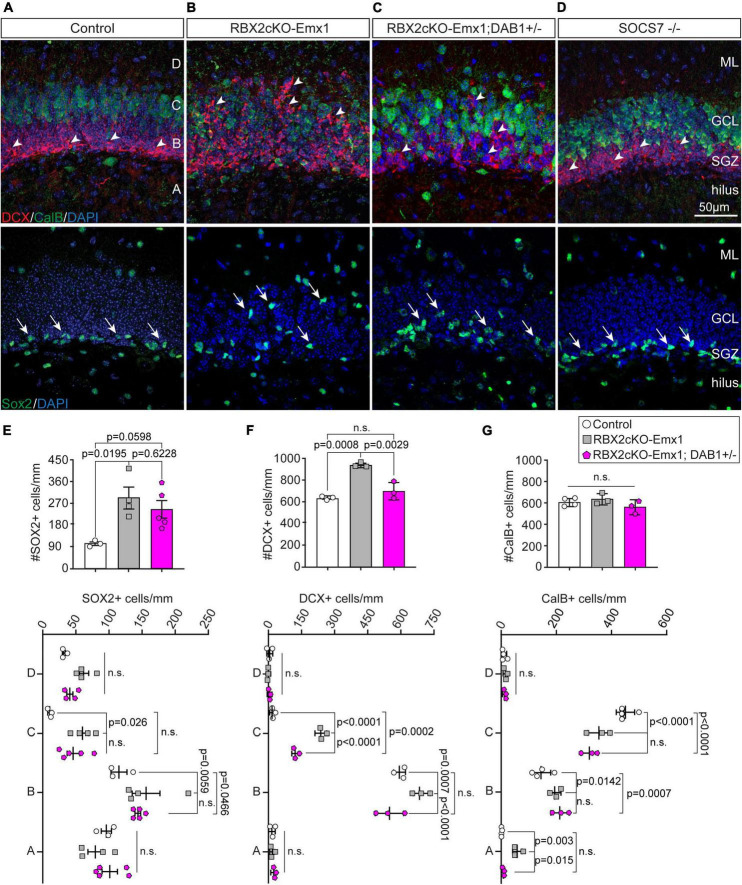
RBX2 regulates layering during DG development and controls NSC proliferation. **(A,B)** Depletion of RBX2 disrupted the localization of Doublecortin (DCX)+ (arrowheads) and calbindin (CalB)+ cells in comparison to control DG at P21. Similarly, SOX2+ NSCs, which normally locate in the subgranular zone (SGZ), were displaced to other areas of the DG upon RBX2 depletion. **(C)** Reducing DAB1 accumulation partially rescued the localization defects of DCX+ cells caused by knocking out RBX2, whereas it had little effect in CalB+ and SOX2+ cells. **(D)** Knocking out SOCS7 did not affect the neuron localization and layering in the DG. Total number (top) and layer distribution (bottom) of SOX2+ **(E)**, DCX+ **(F)**, and CalB+ **(G)** cells in the DG. The DG was divided in four layers as shown in **(A)**. Mean ± SEM. Statistics, one-way ANOVA with Tukey’s method adjusted *p*-value for total number of cell analyses and two-way ANOVA with Tukey’s method adjusted *p*-value for layering analyses. ML, molecular layer; GCL, granule cell layer.

CalB staining also revealed dramatic layering defects in the DG of Rbx2cKO-Emx1 mice (Figure 1A). To further investigate this phenotype, we analyzed the distribution of mature GCs (CalB+), immature GCs/IPs (DCX+), and type-1/2a NSCs (SOX2+) in P21 control and RBX2 mutant DGs (Brown et al., 2003; Hsieh, 2012; Goncalves et al., 2016). As expected from control DGs, the majority of CalB+ somas located in the outer-half of the of granule cell layer (GCL), and the DCX+ cells and SOX2+ NSCs in the subgranular zone (SGZ) (Figures 2A,E–G). In comparison, knocking out RBX2 completely disrupted DG layering with CalB+ neurons located in all layers of the DG, including the hilus, and DCX+ and SOX2+ cells displaced from the SGZ (Figures 2B,E–G).

Given that Reelin/DAB1 signaling is important for DG layering and reducing DAB1 levels partially rescues the layering phenotypes in the RBX2 mutant cortex (Simo and Cooper, 2013), we assessed whether sustained Reelin/DAB1 signaling was also responsible for the DG layering defects observed upon RBX2 depletion. We analyzed the DG layering in Rbx2cKO-Emx1; DAB1 +/– mice, in which pY-DAB1 levels are significantly reduced, and in SOCS7 –/– mice, in which pY-DAB1 accumulates (Supplementary Figure 2). Reducing pY-DAB1 levels partially rescued the misposition of DCX+ cells, whereas it failed to rescue ectopic SOX2+ and CalB+ cells (Figures 2C,F,G). Moreover, accumulation of pY-DAB1, in SOCS7 –/– mice, was not sufficient to cause any layering defects (Figure 2D). Interestingly, knocking down RBX2 and ARF6 worsen the layering phenotypes with further displacement of CalB+ GCs in the molecular layer (ML) and hilus (Supplementary Figures 1D,D’), a phenotype previously observed in hippocampal pyramidal neurons (Han et al., 2020).

**FIGURE 3:**
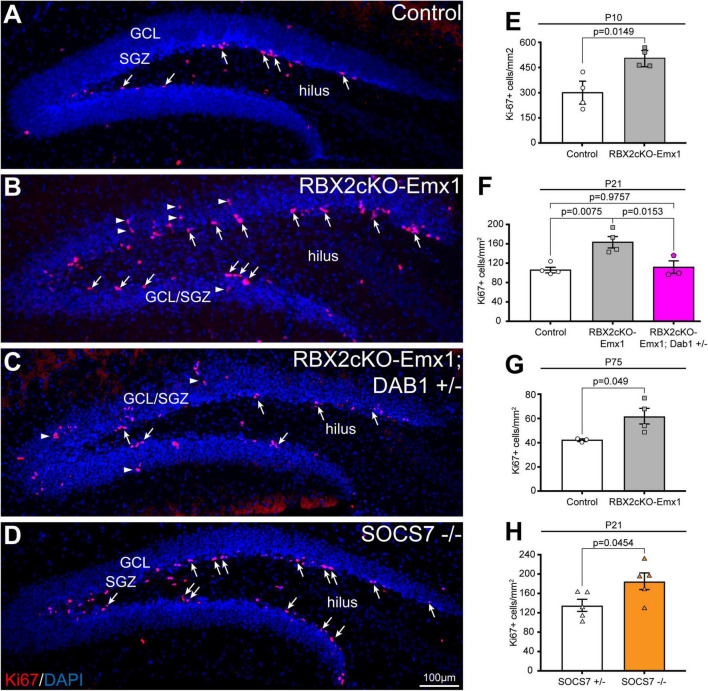
RBX2 controls NSC proliferation in a Reelin/Dab1-dependent fashion. **(A,B)** Knocking out RBX2 promoted NSC proliferation, detected by Ki-67 staining (arrows), in comparison to control DG at P21. Arrowheads indicate Ki-67+ cells away from the innermost layer in the DG **(C)** reducing DAB1 levels in the RBX2 mutant DG rescues cell proliferation. **(D)** SOCS7 depletion showed the same levels of Ki-67+ cells (arrows). **(D–F)** Quantification of Ki-67+ cells in control (*Rbx2 fl/fl*) vs. RBX2cKO-Emx1 DG at P10 **(E)**, P21 also including RBX2cKO-Emx1; DAB1 +/– quantification **(F)**, P75 **(G)**, and of control (SOCS7+/–) and SOCS7 mutant (SOCS7–/–) DG at P21 **(H)**. Mean ± SEM. Statistics, unpaired Student’s *t*-test **(E,G,H)** and one-way ANOVA with Tukey’s method adjusted *p*-value **(F)**.

Importantly, depletion of RBX2 increased the number of SOX2+ and DCX+ cells in the hippocampus in comparison to control (Figures 2E,F). However, only the increment of DCX+ cells was abolished when DAB1 levels were rescued, whereas the number of SOX2+ cells failed to change between control and Rbx2cKO-Emx1; DAB1 +/– (Figure 2F). No differences in the amount of mature granule cells were detected in any condition (Figure 2G). An increased number of DCX+ cells suggests that RBX2 depletion promotes NSC proliferation in the DG. To assess this possibility, we stained control and Rbx2cKO-Emx1 brains with the cell cycle marker Ki-67 (Peissner et al., 1999). In comparison to controls, RBX2 mutant hippocampus showed a significant increase in Ki-67+ cells at P10, P21, and in adult DGs, albeit at progressively lower levels as animals aged (Figures 3A,B,E–G; Kuhn et al., 1996). Given that Reelin/DAB1 signaling promotes adult neurogenesis (Pujadas et al., 2010; Teixeira et al., 2012) and reduction of DAB1 levels rescued the number of DCX+ cells in the RBX2 mutant DG (Figure 2F), we hypothesized that sustained Reelin/DAB1 signaling was responsible for the increased cell proliferation in absence of CRL5 activity. To test this possibility, we analyzed the number of actively proliferating cells in RBX2cKO-Emx1; DAB1 +/– and SOCS7 mutant DGs. Whereas reducing DAB1 levels in the RBX2 mutant hippocampus rescues the number of proliferating cells, depletion of SOCS7 promoted a similar increase in the number of Ki-67+ cells in the DG as observed in RBX2 mutant mice (Figures 3C,D,F,H). Despite the increased proliferation rate, we did not observe an obvious change in DG size between control and RBX2 mutant DG. This prompted us to analyze the survival rate of adult-born GCs in absence of CRL5 activity by injecting the thymidine-analog EdU at P21 in control and RBX2cKO-Emx1 mice and counting the number of EdU+ cells present in the DG after 6 month. We found less EdU+ cells in the RBX2 mutant DG, suggesting either a decrease in GC survival or an increase in NSC proliferation without neurogenesis (e.g., NSC self-renewal) (Supplementary Figure 3A).

We further investigated the direct role of RBX2 promoting NSC proliferation and adult neurogenesis. We crossed our *rbx2* floxed animals with *Nestin-Cre/ERT2* mice, which express tamoxifen-inducible Cre in adult NSCs, and the Cre-reporter mice Ai9 (Lagace et al., 2007; Madisen et al., 2010). Rbx2 fl/fl; Ai9/+; Nestin-Cre/ERT2 or control (control mice had only one *rbx2* allele floxed) littermates were treated with tamoxifen at P30 and brains were collected 30 days post treatment. Surprisingly, no significant differences in the number of tdTomato+ cells were observed between control and RBX2-depleted brains (Supplementary Figure 3B). These data indicates that depletion of RBX2 in NSCs at juvenile stages is not sufficient to promote NSC proliferation and neurogenesis.

### Increased Motor Activity in Rbx2cKO-Emx1 Mice

**FIGURE 4:**
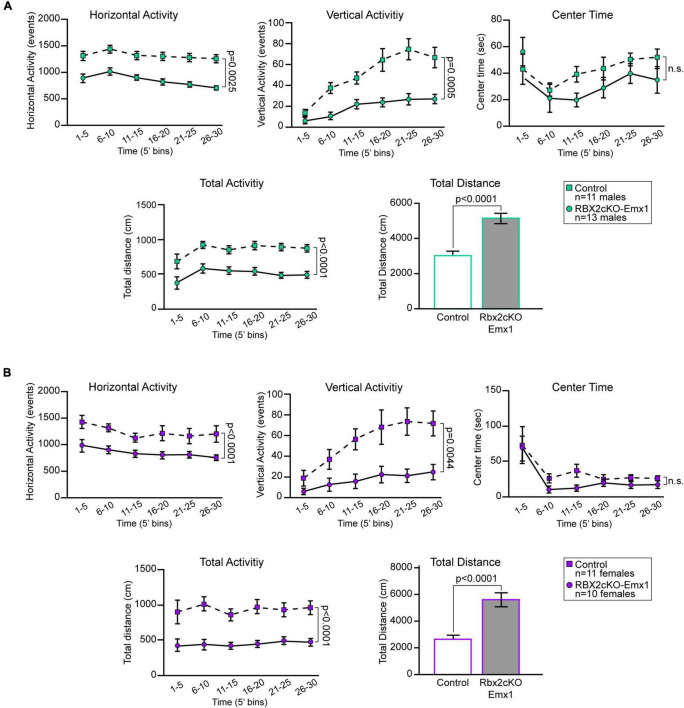
In an open field test, both RBX2 mutant male **(A)** and female **(B)** mice (P75) showed increased horizontal (distance moved) and vertical (rearing) activity as well as higher total activity and distance moved in 30 min in comparison to control mice. On the contrary, both male and female RBX2cKO-Emx1 mice did not show changes in the amount of time spent at the center of the field. Mean ± SEM. Statistics, two-way ANOVA for all quantifications, except unpaired Student’s *t*-test for total distance quantification.

Among the factors that most highly correlate with increased adult neurogenesis is physical exercise (Goncalves et al., 2016; Saraulli et al., 2017). To test whether RBX2 depletion affects mouse behavior, particularly mouse physical activity, we performed open field tests with adult control and Rbx2cKO-Emx1 mice (P75). Depletion of RBX2 significantly increased overall mouse activity (Figure 4). Both horizontal and vertical (i.e., rearing) activity, as well as total distance travel were increased in Rbx2cKO-Emx1 females and males. No differences in the amount of time spent in the center of the field were observed between genotypes or gender (Figure 4), excluding anxiety-related differences. Importantly, the increased activity in RBX2 mutant mice was observed in all time bins analyzed, suggesting that changes in exploratory drive or anxiety, mostly shown during the first minutes of the test, are not the principal drivers for the phenotypes observed (Gould et al., 2009). Overall, our data indicate that CRL5 regulates mouse activity, which in turn may promote the increase in NSC proliferation observed in the adult RBX2 mutant DG.

## Discussion

DG morphogenesis requires the timely activation of myriad of signaling pathways and the interaction of multiple cell types in a coordinated fashion (Frotscher et al., 2003; Li and Pleasure, 2007; Cayre et al., 2009; Li et al., 2009; Sibbe et al., 2009; Wang et al., 2018; Nelson et al., 2020). Whereas much progress has been made in understanding the molecular mechanisms involved in cortical projection neuron migration and cortical layering, fewer studies address the molecular complexity underlying DG development. Moreover, the importance of signal termination for DG morphogenesis or homeostasis, including adult neurogenesis, remains for the most part unknown.

Our results show that CRL5 participates in mossy fibers IPB pruning. Genetic depletion of RBX2, which renders CRL5 complex inactive, disrupts IPB pruning causing IPB overextension. These axonal defects were not related to CRL5-dependent regulation of Reelin/DAB1 or ARF6 signaling, as reducing DAB1 levels or knocking out ARF6 in the context of RBX2 depletion failed to rescue IPB pruning. RBX2 mutant GC axons were capable to respond to Semaphorin-3Y in a common axon repulsion assay (Chen et al., 2000; Cheng et al., 2001), indicating their capability of sensing Sempahorin-3Y through Plexin-A3 and Neuropilin-2 co-receptors and triggering the appropriate signaling pathways despite CRL5 inactivation (Zhou et al., 2008). In comparison to axon repulsion where the signaling cascade is triggered by Plexin-A3, Sempahorin-3Y-dependent mossy fiber pruning initiates at Neuropilin-2 by the recruitment of the RAC GAP β2-Chimaerin and triggering the inhibition of RAC1 (Riccomagno et al., 2012). Interestingly, another molecular mechanism where Ephrin-B3 reverse signaling leads to activation of RAC1 (i.e., higher RAC1-GTP levels) prior IPB pruning has been described (Xu and Henkemeyer, 2009). Our results show that IPB pruning is blocked in absence of CRL5 activity and the levels of RAC1-GTP and CDC42-GTP are lower than control. Thus, our data suggest that CRL5 may participate on IPB pruning in a similar manner as Ephrin-B3 reverse signaling. It is possible that both signaling pathways dial in on RAC1 to tightly regulate its temporal and spatial activation to control IPB pruning. Future work should address whether CRL5 directly participates in EphB/Ephrin-B3 or Semaphorin-3F signaling and determine its molecular involvement.

Similarly to other layered structures in the central nervous system (Simo and Cooper, 2013; Fairchild et al., 2018; Han et al., 2020), CRL5 regulates neuron position and lamination in the DG. Given the complex migration behaviors of the cells forming the DG (Nelson et al., 2020), it is difficult to predict when and where the layering defects observed in the RBX2 mutant DG arise. A riveting possibility is that lack of CRL5 activity impedes DG cells to hold their intended locations and disperse, as previously observed in cortical projection neurons (Simo and Cooper, 2013). On the contrary, the role of sustained Reelin/DAB1 signaling has a smaller contribution in the DG layering in comparison to cortex, given that reducing DAB1 levels mildly rescued GC ectopic layering and SOCS7 mutant DG showed no layering defects. Importantly, depletion of ARF6 exacerbated the neuron dispersion phenotype observed in absence of RBX2, suggesting that ARF6 overactivation impairs GC motility (Falace et al., 2014; Han et al., 2020).

Moreover, we showed that depletion of RBX2 promotes NSC proliferation likely through sustained Reelin/DAB1 signaling, as similar number in Ki-67+ cells are observed in RBX2 and SOCS7 mutant DG. The most likely scenario is that NSC proliferation promotes adult neurogenesis and, supporting this hypothesis, ectopic Reelin over-expression in the adult hippocampus also promotes NSC proliferation and adult neurogenesis (Pujadas et al., 2010). If this hypothesis is correct, decreased adult-born GC survival in the RBX2 mutant DGs is likely a consequence of failing integration into the existing synaptic network due to the DG layering defects observed in these animals (Doengi et al., 2016; Huckleberry and Shansky, 2021). Alternatively, increased NSC proliferation may represent an exuberant form of NSC and IP self-renewal, which would increase the numbers of SOX2+ and DCX+ cells, as observed in the RBX2 mutant DG, and consequently decreasing the number of adult-born GCs.

Surprisingly, knocking out RBX2 in adult Nestin+ cells (i.e., NSCs) did not promote an increase overall cell proliferation suggesting that non-cell autonomous mechanisms are involved to promote NSC/IP proliferation in absence of RBX2 or that RBX2 depletion at two developmental stages (e.g., embryonic -Emx1-Cre- vs. juvenile -Nestin-Cre/ERT2) had differential cellular effects.

We also demonstrate that RBX2 depletion promotes physical activity. Physical activity is well known to promote adult neurogenesis and GC survival in the DG (Vivar et al., 2013; Goncalves et al., 2016; Saraulli et al., 2017). Thus, we hypothesize that CRL5-dependent increase in physical activity may enhance NSC proliferation in the adult DG. Future work should address how CRL5 inactivation in the telencephalon promotes higher levels of physical activity.

## Data Availability Statement

The original contributions presented in this study are included in the article/Supplementary Material, further inquiries can be directed to the corresponding author.

## Ethics Statement

The animal study was reviewed and approved by the Institutional Animal Care and Use Committees (University of California, Davis).

## Author Contributions

AL, ED, and SS designed the research. RR, KH, CC, and SS performed the research. AL and ED contributed to the new reagents and analytical tools. SS wrote the manuscript with AL and ED editing. All authors contributed to the article and approved the submitted version.

## Conflict of Interest

The authors declare that the research was conducted in the absence of any commercial or financial relationships that could be construed as a potential conflict of interest.

## Publisher’s Note

All claims expressed in this article are solely those of the authors and do not necessarily represent those of their affiliated organizations, or those of the publisher, the editors and the reviewers. Any product that may be evaluated in this article, or claim that may be made by its manufacturer, is not guaranteed or endorsed by the publisher.

## Supplementary Material

Supplementary Figure 1 Defects in IPB pruning in RBX2 mutant DG are independent of age and Reelin/Dab1 and ARF6 signaling. **(A)** IPB overextension in the RBX2cKO-Emx1 mice remains in adult mice (P75). Similar to P21 results, CalB stainings of control and RBX2cKO-Emx1 DG showed an ectopically IPB extension only in the RBX2 mutant DG. Arrows and arrowheads demarcate SPB and IPB extension, respectively. **(B)** SOCS7 depletion, which causes a sustained activation of Reelin/DAB1 signaling, does not affect IPB pruning. **(C)** Reducing the accumulation levels of DAB1 in the RBX2 mutant DG is not sufficient to rescue IPB pruning. **(D)** Similarly, knocking out ARF6 together with RBX2 failed to rescue IPB pruning. D’, double RBX2 and ARF6 mutant DG showed an increased dispersion of CalB+ cells.

Supplementary Figure 2 pY-DAB1 and DAB1 accumulation levels in RBX2 and SOCS7 mutant models. **(A)** DAB1 stainings in P21 control (RBX2 fl/fl), RBX2cKO-Emx1, SOCS7–/–, RBX2cKO-Emx1; DAB1+/– DG, and DAB1–/–. **(B,C)** Western blotting of P10 control and Rbx2cKO-Emx1 **(B)** and SOCS7–/– **(C)** hippocampal lysates. Red arrowhead indicates pY-Dab1.

Supplementary Figure 3 **(A)** Decreased number of EdU+ cells survived in the RBX2 mutant DG in comparison to control. Mean ± SEM. Statistics, unpaired Student’s *t*-test. **(B)** Representative images of control and tamoxifen-dependent depletion of RBX2 in NSCs does not promote adult neurogenesis. Mean ± SEM. Statistics, Mann Whitney test.
