# Supplementary figures and images for: The E3 Ubiquitin Ligase CRL5 Regulates Dentate Gyrus Morphogenesis, Adult Neurogenesis, and Animal Behavior
Source: Front Neurosci. 2022 Jun 21;16:908719. doi: 10.3389/fnins.2022.908719 (PMC9253586; doi:10.3389/fnins.2022.908719)

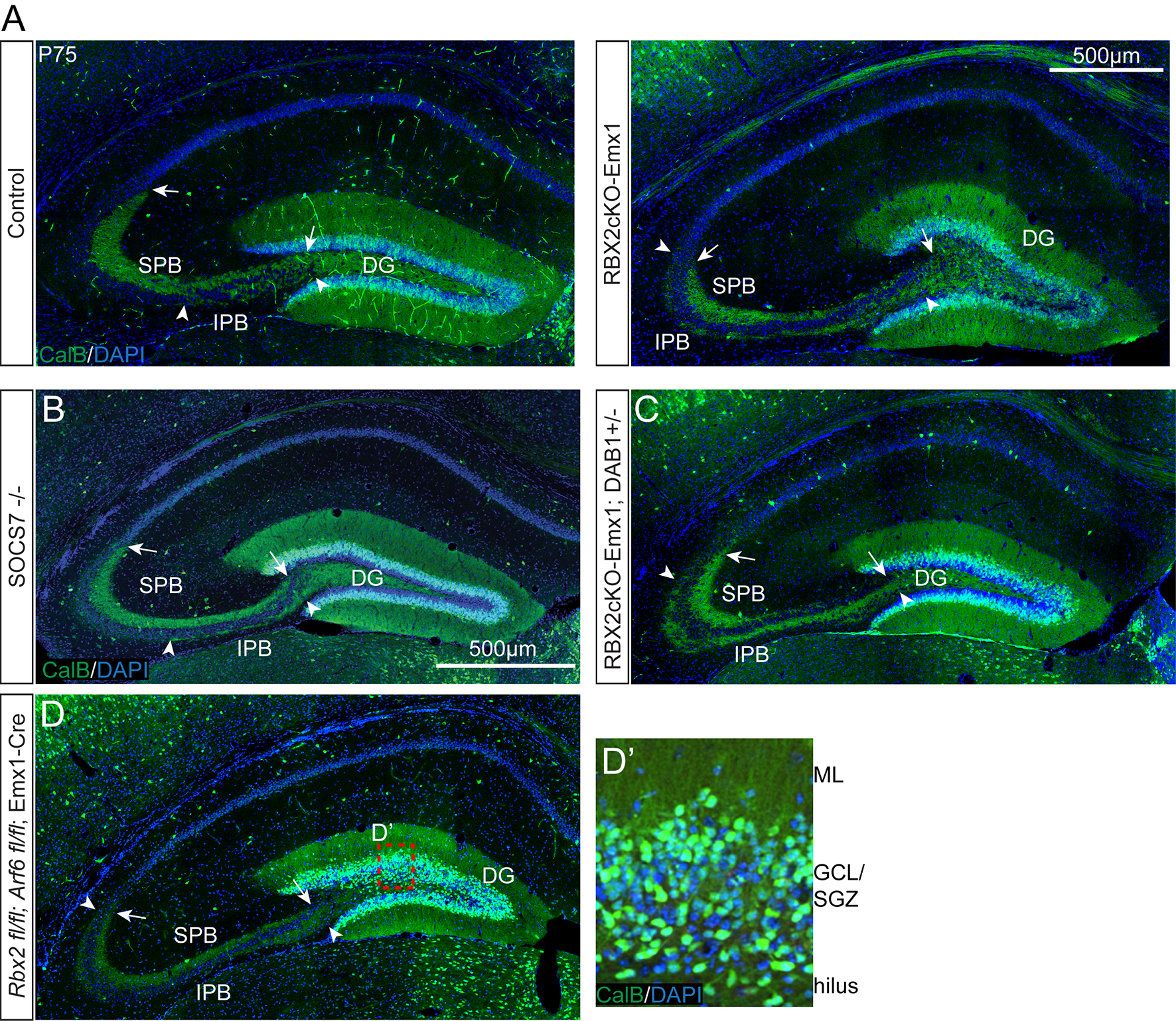

Supplement: Supplementary Figure 1 — Defects in IPB pruning in RBX2 mutant DG are independent of age and Reelin/Dab1 and ARF6 signaling. (A) IPB overextension in the RBX2cKO-Emx1 mice remains in adult mice (P75). Similar to P21 results, CalB stainings of control and RBX2cKO-Emx1 DG showed an ectopically IPB extension only in the RBX2 mutant DG. Arrows and arrowheads demarcate SPB and IPB extension, respectively. (B) SOCS7 depletion, which causes a sustained activation of Reelin/DAB1 signaling, does not affect IPB pruning. (C) Reducing the accumulation levels of DAB1 in the RBX2 mutant DG is not sufficient to rescue IPB pruning. (D) Similarly, knocking out ARF6 together with RBX2 failed to rescue IPB pruning. D’, double RBX2 and ARF6 mutant DG showed an increased dispersion of CalB+ cells. [file Image_1.TIF]

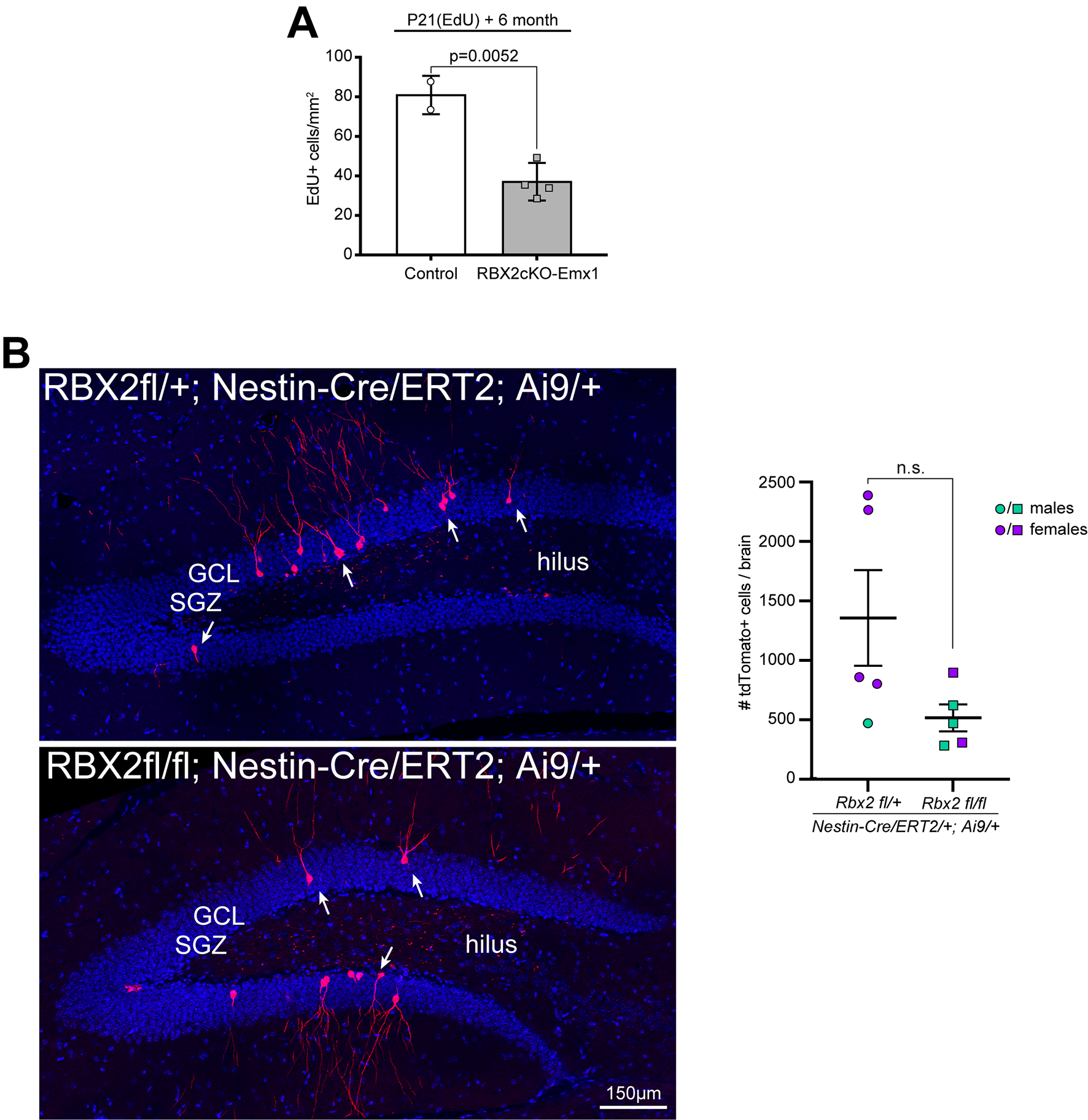

Supplement: Supplementary Figure 2 — pY-DAB1 and DAB1 accumulation levels in RBX2 and SOCS7 mutant models. (A) DAB1 stainings in P21 control (RBX2 fl/fl), RBX2cKO-Emx1, SOCS7–/–, RBX2cKO-Emx1; DAB1+/– DG, and DAB1–/–. (B,C) Western blotting of P10 control and Rbx2cKO-Emx1 (B) and SOCS7–/– (C) hippocampal lysates. Red arrowhead indicates pY-Dab1. [file Image_2.TIF]

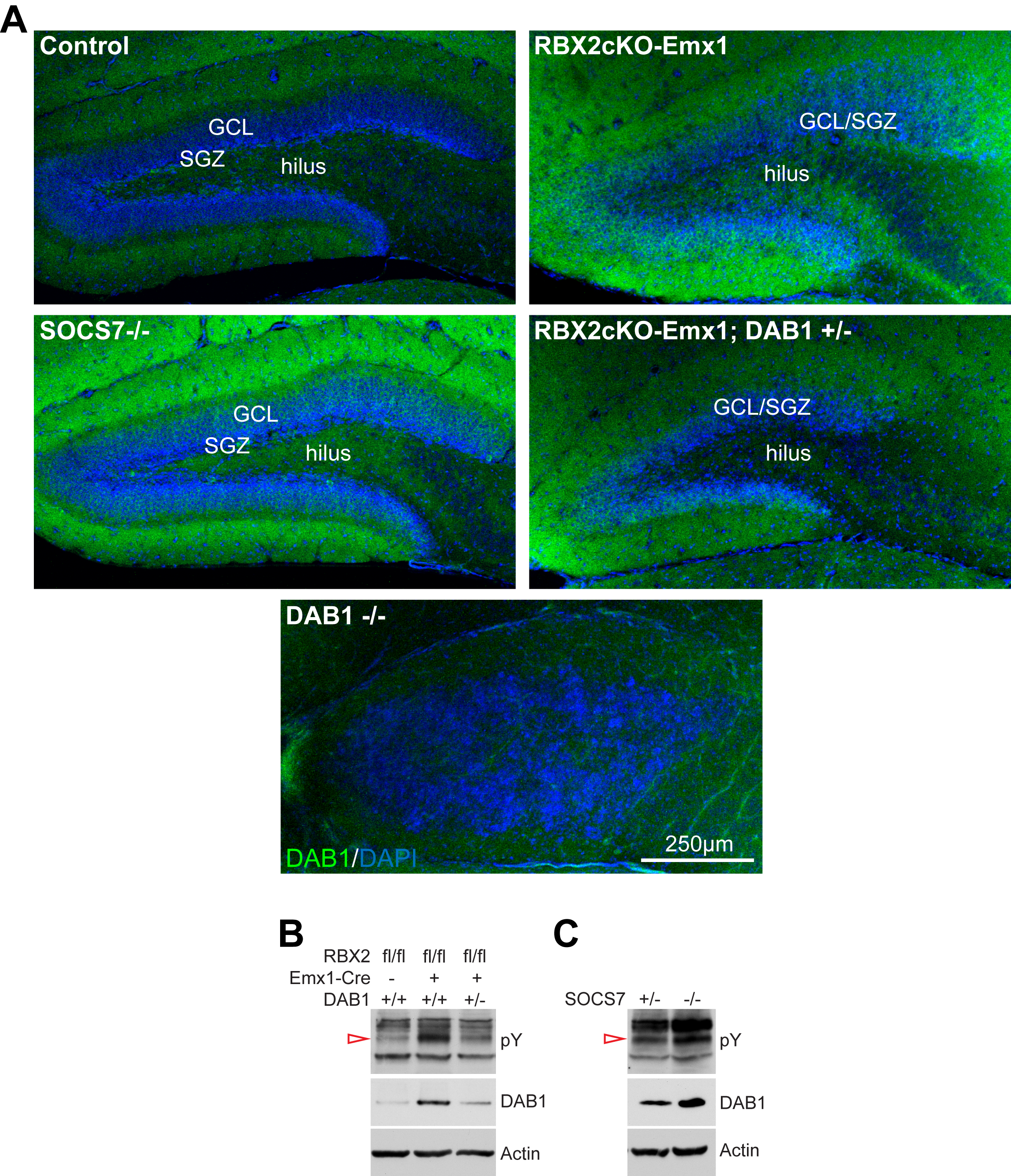

Supplement: Supplementary Figure 3 — (A) Decreased number of EdU+ cells survived in the RBX2 mutant DG in comparison to control. Mean ± SEM. Statistics, unpaired Student’s t-test. (B) Representative images of control and tamoxifen-dependent depletion of RBX2 in NSCs does not promote adult neurogenesis. Mean ± SEM. Statistics, Mann Whitney test. [file Image_3.TIF]
